# Case Report: Successful Management of a Refractory Plasmablastic Lymphoma Patient With Tislelizumab and Lenalidomide

**DOI:** 10.3389/fimmu.2021.702593

**Published:** 2021-07-12

**Authors:** Lili Cheng, Qi Song, Mengke Liu, Yan Wang, Hongmei Yi, Ying Qian, Pengpeng Xu, Shu Cheng, Chaofu Wang, Li Wang, Weili Zhao

**Affiliations:** ^1^ Shanghai Institute of Hematology, State Key Laboratory of Medical Genomics, National Research Center for Translational Medicine at Shanghai, Ruijin Hospital Affiliated to Shanghai Jiao Tong University School of Medicine, Shanghai, China; ^2^ Department of Radiology, Shanghai Rui Jin Hospital, Shanghai Jiao Tong University School of Medicine, Shanghai, China; ^3^ Department of Pathology, Shanghai Rui Jin Hospital, Shanghai Jiao Tong University School of Medicine, Shanghai, China

**Keywords:** immune checkpoint inhibitor, tislelizumab, lenalidomide, Epstein-Barr virus, plasmablastic lymphoma

## Abstract

Plasmablastic lymphoma (PBL) is a rare and aggressive hematological malignancy. PBL commonly occurs in immune incompetent patients, such as those with human immunodeficiency virus (HIV), post-transplant status, or immunosenescence. Given its rarity, there is no specific standard treatment for PBL. However, small case series have shown that intensive chemotherapies combined with anti-myeloma agents such as bortezomib and lenalidomide were effective in treating PBL. Unfortunately, some fragile patients could not tolerate intensive chemotherapeutic regimens, especially the elderly patients. Here we presented a 76-year-old female PBL patient refractory to miniCHOP regimen combined with bortezomib but achieved complete remission when treated with tislelizumab combined with lenalidomide, indicating that immune therapy may be a potential treatment for PBL. To our knowledge, this is the first chemoresistant PBL patient that has been successfully treated with checkpoint inhibitor plus lenalidomide, thus providing new insight towards PBL management.

## Introduction

Plasmablastic lymphoma (PBL) is a rare and aggressive B-cell malignancy first described in 1997 on patients with human immunodeficiency virus (HIV) infection (1). PBL patients frequently experienced immunodeficiencies relating to HIV and immune-suppressive agents for autoimmune diseases or organ transplantation, as well as immunosenescence related to both age and Epstein–Barr virus (EBV) infection (2, 3). EBV-encoded small RNA (EBER) expression has been found in 80% of the HIV-positive cases and 50% of the HIV-negative cases (4). Compared with EBV-negative PBL, EBV-positive PBL displays immune escape patterns with high expression of immune checkpoints such as the programmed cell death protein-1 (PD-1) and its ligand (PD-L1) on tumor cells or in their microenvironment (5). Of note, EBV-positive status is an independent unfavorable factor of PBL (6). Given its rarity, there is no specific standard treatment for PBL. The use of CHOP (cyclophosphamide, doxorubicin, vincristine, and prednisone) and CHOP-like regimens are considered inadequate (7), and more intensive regimens including EPOCH (etoposide, cyclophosphamide, doxorubicin, vincristine, and prednisone), hyper-CVAD (hyperfractionated cyclophosphamide, doxorubicin, vincristine, and dexamethasone), CODOX-M/IVAC (cyclophosphamide, vincristine, doxorubicin, high-dose methotrexate/ifosfamide, etoposide, and high-dose cytarabine) are effective in young or fit patients (4). Despite these intensive chemotherapeutic approaches, additional options beyond chemotherapy are available, such as bortezomib, lenalidomide, brentuximab vedotin, and anti-IL6 and IL6R antibodies (8). Bortezomib plus EPOCH have a complete response rate exceeding 90% as a frontline regimen in sixteen PBL patients (9). B-CHOP (bortezomib plus CHOP) has also been reported to achieve complete and durable remissions in three HIV-positive PBL patients (10). Lenalidomide is an immunomodulatory agent widely used in treating myeloma. Recently, a refractory PBL was successfully treated with lenalidomide in combination with CHOP (11). Due to the high expression pattern of PD-1/PD-L1 on PBL tumor cells and in the microenvironment (12), an immune checkpoint inhibitor might be a potential treatment. Here, we report a chemoresistant PBL patient who achieved complete remission with tislelizumab combined with lenalidomide.

## Case Presentation

**Figure 1:**
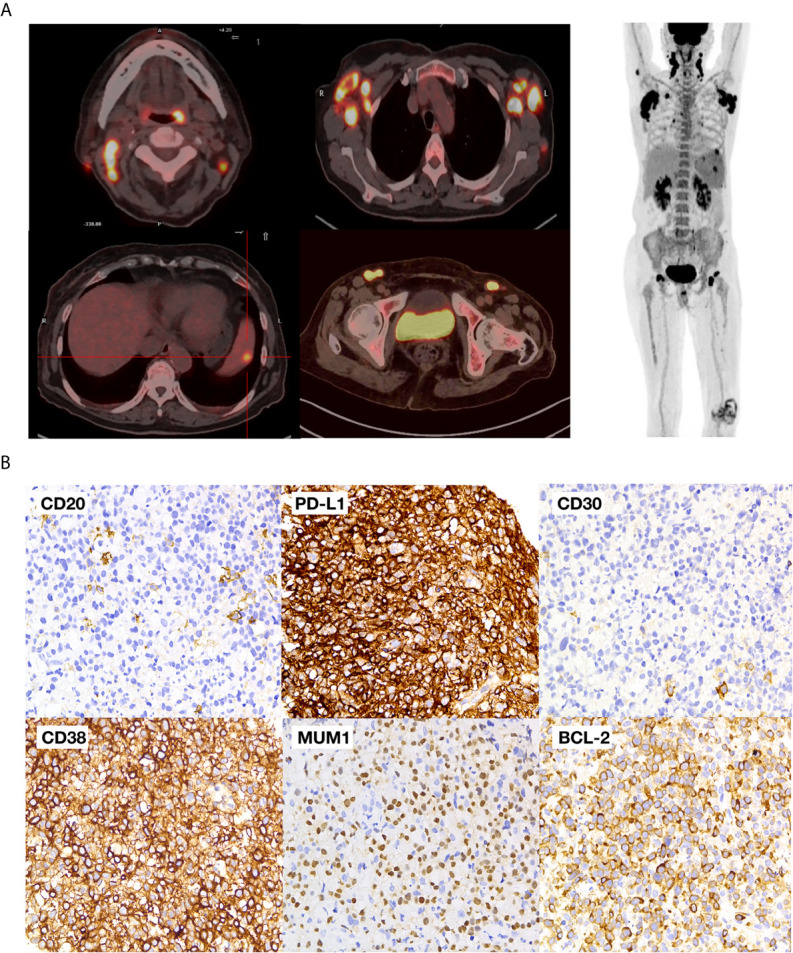
Baseline PET-CT and pathological images **(A)** PET–CT images of the patient; **(B)** pathological images of the tumor.

A 76-year-old female experienced repeated fever accompanied by chills, fatigue, decreased appetite, and shortness of breath for one month. Physical examination on the patient found palpable cervical as well as axillary and inguinal lymph node enlargement. Laboratory work-up showed white blood cells (WBCs) 3.16  ×  10^9^/L, hemoglobin (Hgb) 89g/L platelets 123  ×  10^9^/L, erythrocyte sedimentation rate (ESR) was at 50 mm/h, C-reactive protein at 52 mg/L and serum lactate dehydrogenase at 356 IU/L. Her liver, renal, and thyroid functions were normal. However, serum EBV-DNA was 5,300 copies/ml, and HIV, CMV, hepatitis B, and C serologies were all negative. She was considered to have a putative infection due to high ESR. She underwent ceftazidime treatment but showed no response to antibiotics. Further fluorodeoxyglucose positron emission tomography–computed tomography (PET–CT) indicated multiple lymph node enlargements in the neck, clavicle, axilla, and bilateral inguen with a maximum standard uptake volume (SUV) of 31.2 (**Figure 1A**). A biopsy of the right axillary lymph node showed PBL with tumor cells positive for CD79a, CD38, PD-L1 (90%), MUM-1, EBV-encoded RNA (EBER), BCL-2 (80%), and ki-67 (80%), but negative for CD20, CD30 (**Figure 1B**). Fluorescence *in situ* hybridization (FISH) analysis demonstrated no translocation of MYC, BCL2, and BCL6. The mutation of genes *MYC*, *PRDM1*, *TP53*, and *MYD88*, was not found through the next-gene sequencing on the tumor sample. Bone marrow biopsy showed no marrow involvement.

Considering the advanced age, poor performance status and the international prognostic index (IPI) score of 4, the patient received miniCHOP plus bortezomib [B-miniCHOP, bortezomib 1.3 mg/m^2^ (2.6 mg) day 1, cyclophosphamide 400 mg/m^2^ (815 mg) day 1, liposomal doxorubicin 20 mg day 1, vincristine 1 mg/m^2^ (2 mg) day 1, and prednisone 40 mg/m^2^ (80 mg) on days 1–5] (13). However, the fever and other symptoms did not improve in spite of the induction therapy. One week after the start of B-miniCHOP, lenalidomide 25 mg orally every other day was added. Neither fever nor the size of the enlarged lymph nodes subsided.

**Figure 2:**
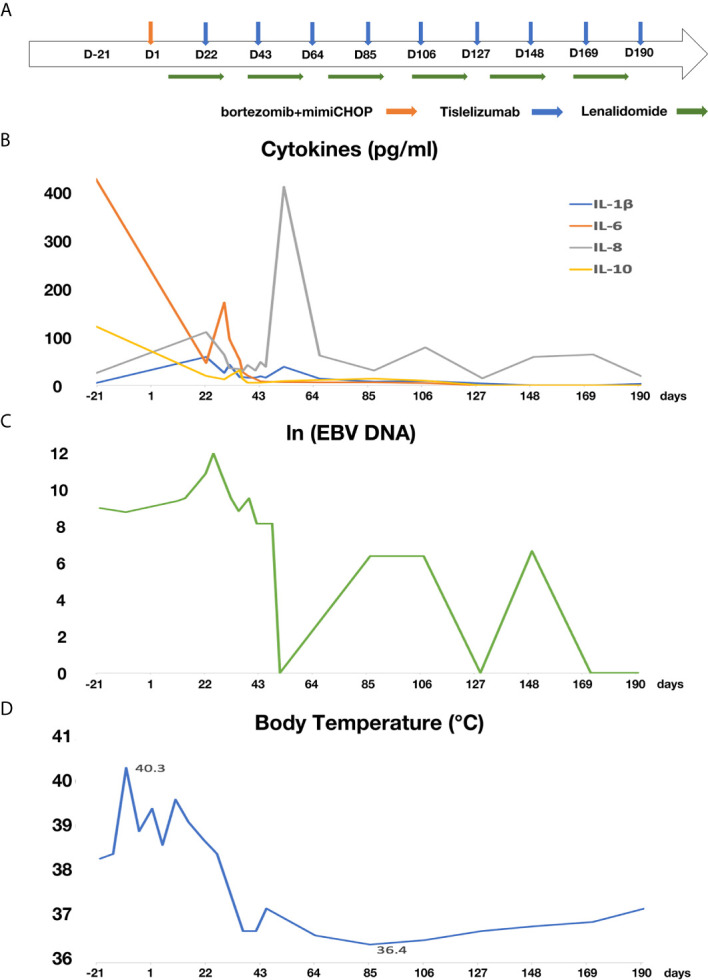
The treatment schedule and the change of cytokines, serum EBV-DNA and body temperature of the patient. **(A)** Flow chart of treatment; **(B)** the change of cytokines; **(C)** the change of EBV-DNA **(D)** the change of body temperature.

**Figure 3:**
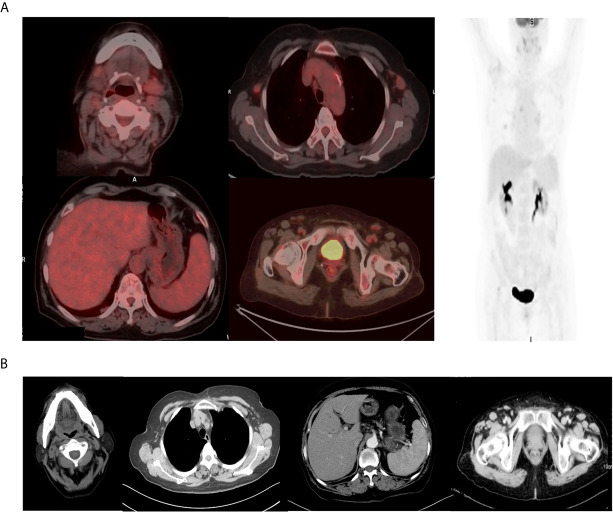
The final PET–CT and enhanced CT evaluation of the patient. **(A)** The final PET–CT evaluation showed complete metabolism remission after eight cycles of tislelizumab and lenalidomide. **(B)** The enhanced CT evaluation showed complete remission 4 months after PET scan.

Two weeks after lenalidomide, anti-PD-1 antibody tislelizumab was initiated at a dose of 200 mg every 3 weeks (**Figure 2A**). The patient experienced pseudo-progression of the axilla lymph node at day 4 following tislelizumab administration. The cytokines presented transient increase (**Figure 2B**), and the patient presented a paroxysmal episode of transient atrial fibrillation (AF). After appropriate therapy, the patient recovered well from AF, and lymph nodes regressed gradually. Afterward, the patient continued her treatment of tislelizumab every 3 weeks, and lenalidomide 25 mg orally every other day for 20 days, followed by rest for 10 days. Moreover, the level of serum EBV-DNA fluctuated throughout the treatment and eventually fell to an undetectable level with the continuation of tislelizumab (**Figure 2C**). The body temperature returned to a normal level after the first cycle of tislelizumab (**Figure 2D**). Meanwhile, the lymph nodes of the patient palpably regressed after the first cycle of tislelizumab plus lenalidomide and had a PET evaluation with Deauville score 4 after four cycles and Deauville score 3 after eight cycles of tislelizumab plus lenalidomide (**Figure 3A**). Enhanced CT scan was performed 4 months after the final PET and confirmed the complete remission status of the patient (**Figure 3B**). The patient had tislelizumab every 3 weeks for one year and lenalidomide for 10 months. But she stopped the treatment due to financial problem, and afterward, she underwent enhanced CT evaluation of disease every 3 months. She remained in CR to date, with overall survival of 18 months.

During the first cycle of tislelizumab combined with lenalidomide, the patient experienced severe adverse events of anemia (Hgb 56 g/L) and thrombocytopenia (plt 2.0  ×  10^9^/L) but recovered in 14 days with supportive care of platelet transfusion and recombinant human thrombopoietin injection. Only mild leukopenia was found from cycle 2 to cycle 8 (WBC range: 2.7–4.6  ×  10^9^/L), and WBC recovered after the suspension of lenalidomide, indicating the leukopenia might be related to lenalidomide rather than tislelizumab. Her liver, kidney, and thyroid functions remained normal during the whole treatment.

## Discussion

Initially described in HIV-positive individuals, PBL is frequently associated with EBV infection (1). Without specific care for PBL, intensive regimen like EPOCH only had 47.4–62.0% CR in fit patients (14). However, some reports of small-scale cases showed anti-PBL activity of non-cytotoxic agents such as bortezomib and lenalidomide (15). The prognosis of PBL patients is generally poor, with a median overall survival (OS) of 6–19 months (4). Therefore, new therapeutic modalities are required to improve the prognosis of PBL patients.

In addition, several studies show a central role of EBV infection in the pathogenesis of PBL (16, 17). The development of EBV-positive lymphoma may be due to the continuous decrease in CD4+ T lymphocyte counts in immunosuppressed patients, which will weaken the antiviral immunity mediated by CD8 + cytotoxic T lymphocytes, resulting in host cells lacking the ability to control the proliferation of EBV-infected B cells (18).

PD-1, an immune checkpoint receptor, interacts with its corresponding ligand PD-L1 and induces immune evasion by inactivating anti-tumor T-cell responses (19). In EBV-positive PBL, high immune cell infiltration and PD-1/PD-L1 expression in the microenvironment suggest that the tumor immune escape strategies may involve T cell dysfunction and exhaustion (5). Thus, targeting the PD-L1/PD-1 pathway may be a potential therapeutic approach for EBV-positive PBL. Anti-PD-1 antibody was successfully used on a refractory PBL patient who was resistant to DA-EPOCH (dose-adjusted etoposide, prednisone, vincristine, cyclophosphamide, and doxorubicin), KRD (carfilzomib, lenalidomide, and low-dose dexamethasone), and ICE (ifosfamide, carboplatin, and etoposide). Finally, the patient was able to achieve partial response after 16 cycles of nivolumab (20). In our case, the patient had PD-L1 overexpressed on the tumor cells, with positive serum EBV, and responded well to the checkpoint inhibitor, tislelizumab. Of note, at the beginning of tislelisumab, inflammatory factors such as IL-10, IL-6, IL-8, and IL-1β temporarily increased, which might induce dysfunction of organs (21–23). In our case, the patient experienced transient atrial fibrillation shortly after the increase of cytokines. During the process of PD-1 treatment, the level of serum EBV-DNA fluctuated, which is consistent with the report indicating PD-1 antibody could activate PD-1-positive T cells to eliminate EBV-infected cells, and PD-1 maintenance treatment should be continued to control the EBV infection (24). With the treatment of tislelizumab plus lenalidomide, the elderly chemoresistant PBL patient achieved a complete remission and remained in CR to date.

To our knowledge, our case is the first chemoresistant PBL patient who has been successfully treated with checkpoint inhibitor plus lenalidomide, providing new insights for the management of PBL. Prospective clinical trials are warranted to draw definite conclusions.

## Data Availability Statement

The raw data supporting the conclusions of this article will be made available by the authors, without undue reservation.

## Ethics Statement

The study was performed in accordance with the Declaration of Helsinki and the protocol was approved by the Ethics Committee of Shanghai Rui Jin Hospital. The patients/participants provided their written informed consent to participate in this study. Written informed consent was obtained from the individual(s) for the publication of any potentially identifiable images or data included in this article.

## Author Contributions

WZ and LW designed the research study. HY and CW performed the pathological analysis. LC, QS, ML, and YW collected the data. YQ, PX, and SC provided the patients. WZ and LW wrote the paper. All authors contributed to the article and approved the submitted version.

## Funding

This study was supported by research funding from the National Natural Science Foundation of China (81830007, and 81770205), Chang Jiang Scholars Program, Shanghai Municipal Education Commission Gaofeng Clinical Medicine Grant Support (20152206 and 20152208), Clinical Research Plan of SHDC (16CR2017A), Multicenter Clinical Research Project by Shanghai Jiao Tong University School of Medicine (DLY201601), Collaborative Innovation Center of Systems Biomedicine, and the Samuel Waxman Cancer Research Foundation.

## Conflict of Interest

The authors declare that the research was conducted in the absence of any commercial or financial relationships that could be construed as a potential conflict of interest.
